# Childhood adversity and disordered eating: the mediator role of food reward drive

**DOI:** 10.3389/fpsyg.2026.1863415

**Published:** 2026-08-03

**Authors:** Cinzia C. Pocol, Andrei Ion, Andrei C. Miu

**Affiliations:** 1 Cognitive Neuroscience Laboratory, Department of Psychology, Faculty of Psychology and Sciences of Education, Babeș-Bolyai University, Cluj-Napoca, Romania; 2 Assessment and Individual Differences – AID Lab, Department of Psychology and Cognitive Science, University of Bucharest, Bucharest, Romania; 3 Center of Excellence for Neuroscience and Mental Health, Faculty of Psychology and Sciences of Education, Babeș-Bolyai University, Cluj-Napoca, Romania

**Keywords:** childhood maltreatment, disordered eating, eating disorders, reward, stress

## Abstract

**Introduction:**

Disordered eating is a subclinical constellation of symptoms—including uncontrolled eating, emotional eating, and cognitive restraint—that constitutes an important risk factor for eating disorders. We examined the associations between childhood maltreatment and disordered eating symptoms and whether food reward drive is a mediator in this relationship. Domain-general anticipatory pleasure was examined as an alternative mediator.

**Methods:**

Romanian adults (*N* = 377; 85.4% women; mean age = 27.19 years) completed a retrospective assessment of childhood maltreatment at baseline, and 1 month later, they were assessed for food reward drive, domain-general anticipatory pleasure, and disordered eating symptoms.

**Results:**

Emotional abuse and emotional neglect uniquely predicted higher food reward drive. This, in turn, predicted uncontrolled eating, emotional eating, and cognitive restraint, yielding significant indirect effects for both types of maltreatment. After covariate adjustment, only the indirect pathway from emotional neglect to disordered eating via food reward drive remained significant. The results were replicated in a sensitivity analysis excluding participants with potentially biased (minimized) maltreatment reports, in which emotional neglect was the only domain showing a significant indirect effect. Symptom-specific models indicated the strongest pathways for uncontrolled and emotional eating, with weaker effects for cognitive restraint. Domain-general anticipatory pleasure showed an additional, smaller indirect effect in the opposite direction for emotional neglect.

**Discussion:**

These findings highlight food-specific reward drive as a mechanism linking childhood emotional neglect to disordered eating in adults.

## Introduction

Childhood maltreatment has been consistently associated with more than a twofold increase in the risk of eating disorders (Amiri and Sabzehparvar, 2025). Eating disorders rarely emerge abruptly; rather, the pathway from early adversity to clinical pathology appears to involve subclinical symptoms, such as emotional eating, that may gradually escalate into a clinical eating disorder (Rossi and Mannarini, 2025). Therefore, recent research has increasingly focused on these subclinical symptoms (Martin and Strodl, 2023), which do not meet the DSM-5 criteria for an eating disorder but nevertheless constitute an important risk factor for the subsequent development of clinical pathology (American Psychiatric Association, 2013; Nagata et al., 2018; Strodl and Wylie, 2020). These symptoms include emotional eating (overeating in response to negative emotions), uncontrolled eating (overeating accompanied by a sense of loss of control), and cognitive restraint (the conscious restriction of food intake without compromising overall energy balance) (Dawson et al., 2022; Rossi et al., 2024). What remains poorly understood are the precise psychological mechanisms that drive this progression—from early adversity to a subclinical disordered eating and, ultimately, to a clinical eating disorder. One promising candidate mechanism linking childhood maltreatment to disordered eating symptoms is impaired reward processing (Banica et al., 2022).

Reward processing is a multidimensional construct conceptualized in various ways. A common distinction across frameworks is made between anticipatory pleasure, the motivation to seek reward (‘wanting’), and consummatory pleasure, the hedonic response to receiving it (‘liking’) (Dolan et al., 2022). Carver and White's (1994) Behavioral Activation System (BAS) identifies three behavioral dimensions: Reward Responsiveness (the intensity of positive reactions to rewards), Drive (persistence in pursuing goals), and Fun Seeking (the impulsive pursuit of novel rewards). In contrast, the Research Domain Criteria (RDoC) Positive Valence System, grounded in neural mechanisms, identifies three constructs: Reward Responsiveness (hedonic response across anticipation, receipt, and satiation), Reward Learning (learning cues that predict reward), and Reward Valuation (estimating reward probability and magnitude) (National Institute of Mental Health, 2016; Carcone and Ruocco, 2017; Olino et al., 2018). The two frameworks are complementary rather than competing. While they partially overlap in the dimensions they identify, they operate at different levels of analysis. Neurobiological and behavioral studies confirm that these dimensions are distinct at the neural level and are differentially associated with psychopathology (Treadway and Zald, 2013; Smith et al., 2016; Cléry-Melin et al., 2019). For example, childhood interpersonal trauma is linked to reduced Drive but not to Reward Responsiveness or Fun Seeking (Miu et al., 2017), and meta-analytic evidence suggests the strongest trauma-related deficits occur in Reward Learning compared to Reward Responsiveness or Reward Valuation (Oltean et al., 2023). Therefore, precise, dimension-specific assessment of reward processing is critical.

Most research on reward processing has relied on domain-general measures, which assess overall reward sensitivity that are not specific to any particular type of reward. These measures are most commonly operationalized through monetary or social paradigms. However, a growing body of evidence suggests that reward processing may vary as a function of reward type, with results differing depending on the incentive used. Primary rewards (e.g., food) elicit innate responses tied to survival, whereas secondary rewards (e.g., money or social approval) depend on learned associations (Delgado et al., 2008). Indeed, electroencephalogram (EEG) event-related potential (ERP) studies show only modest correlations between neural responses to food and monetary rewards and no correlation between food and social rewards (Kringelbach, 2015; Banica et al., 2022). Consistent with this finding, depressive symptoms have been associated with a heightened approach to food rewards but reduced approach to monetary and social incentives (Fussner et al., 2018; Piccolo et al., 2019; Banica et al., 2022). Notably, existing meta-analyses of childhood maltreatment and reward processing have not included any studies examining food rewards (Oltean et al., 2023; Ruge et al., 2024), and we are not aware of any such studies. Therefore, a direct examination of the association between childhood maltreatment and responses to food reward is warranted.

What is the evidence for food reward processing in disordered eating and other eating-related conditions? Behavioral and neurophysiological studies of disordered eating suggest that these symptoms are linked to heightened—rather than reduced—responsiveness to food rewards (Bado et al., 2019; Banica et al., 2022). Drive and Reward Responsiveness toward food correlate positively with uncontrolled eating in overweight individuals (Bado et al., 2019), and both emotional eating and cognitive restraint have been linked to heightened food-reward sensitivity (Burger and Stice, 2011; Avena et al., 2013; Stapleton and Whitehead, 2014; Jonker et al., 2018; Sutton et al., 2022).

We propose that this pathway reflects the role of food as a coping resource in the context of early emotional adversity: In the absence of adequate caregiver support for stress regulation, children may develop emotion-focused coping strategies, such as consuming rewarding foods to manage negative affect, and repeated use of food for coping may consolidate into elevated food reward drive in adulthood (Kazmierski et al., 2022; Duffy et al., 2018). This is consistent with evidence that emotion dysregulation mediates the relationship between childhood emotional maltreatment and eating psychopathology (Moulton et al., 2015). Food reward drive may, therefore, represent a specific downstream mechanism through which early emotional adversity increases vulnerability to disordered eating in adulthood.

The present study examines whether and how a history of childhood maltreatment relates to disordered eating symptoms and whether this relationship is mediated by food reward drive. Guided by the above reasoning, we hypothesized that childhood maltreatment would predict increased food reward drive, which, in turn, would be most strongly associated with emotional eating and uncontrolled eating. To evaluate the specificity of reward-related mechanisms, we also assessed domain-general anticipatory pleasure and examined whether observed associations were specific to food-related reward processes rather than reflecting broader anticipatory pleasure. In addition, we tested these pathways across three distinct disordered eating symptom domains—uncontrolled eating, emotional eating, and cognitive restraint—to determine whether any indirect effects were specific to symptom categories.

## Materials and methods

### Participants

Data were collected anonymously from Romanian adults. The inclusion criteria were minimal, reflecting the general population scope of the study: Native Romanian speakers aged 18 years or older who completed the assessment battery in full were included, and incomplete submissions were excluded. An *a priori* Monte Carlo power analysis was conducted in RStudio (R Core Team, 2023) to determine the minimum sample size required to detect a moderate indirect effect in the hypothesized simple mediation model. The indirect effect was defined as the product of the *a* path and the *b* path. Moderate effect sizes were specified for planning purposes (*a* = 0.30, *b* = 0.30), with a small direct effect (*c*′ = 0.10). Power was evaluated at *α* = 0.05 using non-parametric percentile bootstrap confidence intervals for the indirect effect. For each candidate sample size, 1,000 Monte Carlo datasets were generated, and within each dataset, the indirect effect was estimated using 1,000 bootstrap resamples. Statistical power was defined as the proportion of simulated datasets in which the 95% bootstrap confidence interval for the indirect effect did not include zero. The minimum sample size required to achieve 80% power under these assumptions was *N* = 116.

We recruited participants online via snowball sampling: invitations were posted in social media groups, shared through a targeted campaign, and emailed to students at several universities. Psychology students at our university received course credit for participation, while other respondents were entered into a draw for two €100 gift cards. Each participant was assigned a unique code to ensure anonymity. All participants provided written informed consent in line with the Declaration of Helsinki after being informed of the study’s aims, procedures, and their rights.

### Procedure

Data were collected across three waves as part of a panel design: T1, T2, and T3. At T1, participants provided sociodemographic and biomedical history information and completed the Childhood Trauma Questionnaire-Short Form (CTQ-SF). Since the CTQ-SF retrospectively assesses objective life history events whose recall remains stable over time (Bernstein and Fink, 1998; Badenes-Ribera et al., 2024), it was deliberately administered before the reward and eating measures to minimize potential priming effects on subsequent responses. At T2, which was scheduled 1 month later, participants completed measures of food reward drive [Reward-Based Eating Drive Scale (RED)], disordered eating [Three-Factor Eating Questionnaire (TFEQ)], and domain-general anticipatory pleasure [Temporal Experience of Pleasure Scale (TEPS)]. At T3, approximately 2 months after T2, participants completed the same measures; the 2-month interval was considered appropriate to allow for meaningful variation while limiting dropout. However, due to substantial attrition at T3, only T2 data were used in the primary analyses. Reminder emails and SMS messages were sent between waves using contact information provided at T1.

### Measures

As no Romanian validations are currently available for the measures used in this study (CTQ-SF, RED, TFEQ, and TEPS), all instruments were translated using standard forward–backward translation procedures to ensure linguistic and conceptual equivalence. Psychometric properties were evaluated in the present sample and are reported for each measure below.

*Sociodemographic and medical history.* Participants reported their age (years), sex, subjective socioeconomic status (SES) (at age 12 and currently; 10-point Likert scale), body mass index (BMI), smoking status (yes/no), current medication use (*Are you currently taking any medication?*; yes/no), alcohol use (*Have there been any periods in your life when you consumed more than five alcoholic drinks (beer, wine, or spirits) on a single occasion?*; yes/no), current medical conditions (*Are you currently diagnosed with any medical conditions, such as chronic illnesses, neurological conditions, or endocrine conditions?*; yes/no), and mental health conditions (*Are you currently diagnosed with a mental health condition by a clinical psychologist or a psychiatrist?*; yes/no).

*Childhood maltreatment*. Childhood maltreatment was assessed using the Childhood Trauma Questionnaire-Short Form (CTQ-SF; Bernstein and Fink, 1998; Bernstein et al., 2003). This self-reported measure comprises 28 items rated on a five-point Likert scale (1 = never true to 5 = very often true) and retrospectively evaluates experiences of abuse and neglect occurring before the age of 18. The CTQ-SF assesses multiple dimensions of maltreatment: (1) emotional abuse (recurrent criticism, humiliation, or psychological hostility); (2) sexual abuse (sexual experiences imposed by an older person); (3) physical abuse (use of physical force with potential for injury); (4) emotional neglect (limited emotional responsiveness or support); (5) physical neglect (inadequate provision of basic needs); and (6) minimization/denial, a validity scale reflecting underreporting or normalization of childhood adversity. Subscale scores were summed, with higher values indicating greater severity. The CTQ-SF demonstrated good to excellent internal consistency across its subscales in our sample: emotional abuse (*α* = 0.85), physical abuse (*α* = 0.79), sexual abuse (*α* = 0.88), emotional neglect (*α* = 0.83), minimization/denial (*α* = 0.82), and physical neglect (*α* = 0.63). The slightly lower alpha value for the physical neglect subscale is consistent with previously reported findings (Badenes-Ribera et al., 2024).

*Food reward drive.* The 13-item Reward-Based Eating Drive Scale (RED) evaluates how strongly eating behavior is influenced by reward processes, such as heightened reactivity to palatable foods, persistent food-related thoughts, and reduced awareness of satiety cues. Items are rated on a five-point Likert scale (0 = strongly disagree to 4 = strongly agree; Vainik et al., 2019), with higher scores indicating stronger reward-driven motivation to eat rather than physiological hunger. In the present sample, the scale demonstrated excellent internal consistency (Cronbach’s *α* = 0.94).

*Domain-General Anticipatory Pleasure.* Anticipatory pleasure was assessed using the Temporal Experience of Pleasure Scale (TEPS; Gard et al., 2006), a 19-item self-report measure rated on a six-point Likert scale. The scale comprises two subscales: Anticipatory pleasure (nine items) and consummatory pleasure (10 items), with higher scores indicating greater hedonic capacity. Only the TEPS anticipatory pleasure was used in this study. The TEPS anticipatory pleasure subscale demonstrated acceptable internal consistency in the present sample (*α* = 0.61).

*Disordered eating.* The Three-Factor Eating Questionnaire–R18 (TFEQ; Anglé et al., 2009) assesses three fundamental patterns of dysregulated eating behavior: (1) cognitive restraint, reflecting intentional efforts to monitor and limit food intake; (2) uncontrolled eating, reflecting difficulties in maintaining control over eating once initiated; and (3) emotional eating, reflecting the use of food as a strategy for coping with negative affect. The questionnaire comprises 18 items rated on four-point response scales (with one item rated on an eight-point scale). Mean scores were calculated for each subscale, with higher values indicating greater levels of maladaptive eating behavior. In this study, the internal consistency estimates, measured by Cronbach’s alpha, ranged from 0.88 to 0.91 across the subscales.

### Statistical analysis

Statistical analysis was conducted using IBM SPSS Statistics (version 29.0; IBM Corp., 2022). Mediation analyses were performed using the PROCESS macro (version 4.0; Hayes, 2022) in SPSS.

Descriptive statistics for the main variables (CTQ subscales, the RED, TFEQ subscale scores, and the TEPS) were summarized and inspected for distributional characteristics, including means, standard deviations, skewness, and kurtosis (see Table 1). CTQ minimization/denial scores were examined as a potential indicator of underreporting by comparing the scores across CTQ subscales between participants with zero and non-zero minimization/denial scores. If underreporting was supported, all primary analyses were complemented by sensitivity analyses restricted to participants with zero scores on the CTQ minimization/denial subscale. Bivariate associations among the main study variables were examined via correlations. Correlations were computed among the five CTQ subscales, RED, TEPS anticipatory pleasure, and TFEQ subscales (uncontrolled eating, emotional eating, and cognitive restraint) to examine the pattern of associations across all primary study variables.

**Table 1:** Childhood maltreatment, food reward drive, domain-general anticipatory pleasure, and disordered eating symptoms.

No.	Variable	Mean ± SEM	Skewness	Kurtosis	1	2	3	4	5	6	7	8	9	10
1	CTQ emotional abuse	11.0 ± 0.27	0.74	−0.35										
2	CTQ physical abuse	6.98 ± 0.18	2.52	7.10	0.55^***^									
3	CTQ sexual abuse	6.1 ± 0.16	3.76	15.07	0.3^***^	0.31^***^								
4	CTQ emotional neglect	8.95 ± 0.15	0.12	−1.23	0.49^***^	0.19^***^	0.13^*^							
5	CTQ physical neglect	6.57 ± 0.12	1.99	4.50	0.57^***^	0.56^***^	0.18^***^	0.38^***^						
6	CTQ total	39.60 ± 0.63	1.13	1.46	0.88^***^	0.74^***^	0.54^***^	0.61^***^	0.72^***^					
7	RED	28.7 ± 0.56	0.81	−0.01	0.2^***^	0.12^*^	0.03	0.19^***^	0.08	0.19^***^				
8	TEPS anticipatory pleasure	31.84 ± 0.33	−0.15	−0.20	−0.03	0.03	−0.02	−0.17^***^	−0.04	−0.06	0.27^***^			
9	TFEQ cognitive restraint	15.64 ± 0.27	−0.03	−0.81	−0.04	−0.08	−0.01	0.0	−0.04	−0.05	0.20^***^	−0.00		
10	TFEQ uncontrolled eating	17.74 ± 0.3	0.68	−0.23	0.21^***^	0.12^*^	0.04	0.21^***^	0.13^*^	0.21^***^	0.86^***^	0.23^***^	0.19^***^	
11	TFEQ emotional eating	6.18 ± 0.15	0.59	−0.79	0.19^***^	0.11^*^	0.05	0.19^***^	0.13^*^	0.19^***^	0.61^***^	0.19^***^	0.23^***^	0.64^***^

Values are reported as mean ± SEM. Correlations are Pearson product–moment correlations shown in the lower triangle. Significance levels are indicated as ^*^p < 0.05, ^**^p < 0.01, ^***^p < 0.001. CTQ, Childhood Trauma Questionnaire; RED, Reward-Based Eating Drive Scale; TEPS, Temporal Experience of Pleasure Scale; TFEQ, Three-Factor Eating Questionnaire.

The primary hypotheses were tested using path analysis with a single mediator, implemented via ordinary least squares regression. Three separate mediation models were estimated, one for each dimension of disordered eating as the outcome: uncontrolled eating, emotional eating, and cognitive restraint. In each model, the five CTQ subscales were entered simultaneously as predictors, with the RED specified as the mediator.

For each model, the *a* paths were estimated by regressing the RED on all five CTQ subscales simultaneously. This approach allowed us to determine the unique association between each CTQ subscale and the RED while controlling for the other CTQ domains. The *b* paths and direct effects (*c* ′ paths) were estimated in a single model by regressing each TFEQ subscale on the RED and all five CTQ subscales simultaneously. Total effects (c paths) were estimated in a separate regression model predicting each TFEQ subscale from the five CTQ subscales without including the RED. Indirect effects for each CTQ subscale were computed as the product of coefficients (a × b) and were evaluated using non-parametric bootstrap confidence intervals with 5,000 resamples. Indirect effects were considered statistically significant when the bootstrap 95% confidence interval did not include zero (Shrout and Bolger, 2002). Multicollinearity was evaluated using variance inflation factors (VIFs) and considered non-problematic when VIFs were below 5 (James et al., 2013).

To assess the robustness of the findings against potential confounding factors, the mediation models were re-estimated with covariate adjustment. Covariates were selected based on their established associations with childhood maltreatment, reward processing, or disordered eating in previous literature. These covariates included age, sex (Alhaj et al. 2022; Soares et al., 2020), socioeconomic status at age 12 and currently (Zielinski, 2009; Austin et al., 2020), BMI, smoking status, substance use, alcohol use, current medication (Poulton and Hester, 2020; Alhaj et al., 2022), and current medical and mental health conditions (Soares et al., 2020).

Childhood food insecurity—defined as reduced access to sufficient food due to limited financial resources (Chilton et al., 2015)—was not introduced as a covariate, regardless of its associations with adverse childhood experiences and disordered eating. Food insecurity is conceptually distinct from the construct of interest: Elevated food reward drive is hypothesized to arise from using food as an emotion regulation strategy in the context of emotional adversity and may be most pronounced in individuals who had sufficient or even abundant access to palatable foods.

Finally, a sensitivity analysis was conducted in the subsample with CTQ minimization/denial scores of zero to reduce the influence of potential maltreatment underreporting. Furthermore, three path analysis models with a single mediator were re-estimated in this subsample—one for each TFEQ subscale (uncontrolled eating, emotional eating, and cognitive restraint)—with the five CTQ subscales as predictors and the RED as the mediator, and indirect effects were again evaluated using 5,000 bootstrap resamples.

## Results

### Sample description

We assessed attrition across the three waves. Compared to T1 (*N* = 643), there was 41.4% attrition at T2 (*N* = 377). T3 included 293 participants (22.3% attrition from T2; 45.6% retention from T1). To assess whether attrition at T3 introduced systematic bias, T3 completers (*n* = 293) and non-completers (*n* = 84) were compared on all key T2 variables using independent samples *t*-tests (see Supplementary Table 6). The groups did not differ significantly on the majority of variables, with two exceptions: T3 completers reported higher emotional neglect and lower cognitive restraint than non-completers, both of which were small in magnitude and are unlikely to have substantially biased the primary analyses. In addition, T2 and T3 scores were strongly correlated across outcome measures (RED: *r* = 0.82; TFEQ: *r* = 0.82; TEPS: *r* = 0.70; all *ps* < 0.001), indicating high temporal stability and suggesting that T2 scores are representative of the construct levels assessed at T3. Overall, to preserve statistical power and minimize potential attrition-related bias, and given the high temporal stability of the measures, the primary analyses were conducted using T1 and T2 data (*N* = 377). Sociodemographic and biomedical characteristics are presented in Table 2.

**Table 2:** Sociodemographic and biomedical characteristics.

Variable	*N*	Statistic	Skewness	Kurtosis
Age (years)	376	27.19 ± 0.57	1.41	1.06
Sex (women)	377	322 (85.4%)	–	–
SES (12 years)	377	6.59 ± 0.09	−0.62	0.29
SES (currently)	377	7.42 ± 0.07	−1.07	2.02
BMI	377	23.17 ± 0.24	1.26	2.05
Smoking (yes)	377	103 (27.3%)	–	–
Current medication (yes)	377	85 (22.5%)	–	–
Alcohol use	377	173 (45.9%)	–	–
Medical conditions (yes)	377	78 (20.7%)	–	–
Mental health conditions (yes)	377	19 (5.0%)	–	–

One participant did not report age. Continuous variables are reported as mean ± standard error of the mean. Categorical variables are reported as *n* (%) for the specified category. Skewness and kurtosis are reported for continuous variables only. BMI, body mass index; SES, socioeconomic status.

Table 1 presents descriptive statistics for all main variables. CTQ subscale scores were positively skewed, particularly for physical abuse, sexual abuse, and physical neglect, consistent with generally low endorsement and a smaller subset of participants reporting higher levels of maltreatment. Based on suggested CTQ cutoffs (see Supplementary Table 1), mean levels fell within the low-to-moderate range for emotional abuse and sexual abuse and within the none-to-minimal range for physical abuse, emotional neglect, and physical neglect. On the CTQ minimization/denial subscale, 101 participants (26.79%) reported non-zero scores, suggesting potential underreporting of maltreatment experiences. Consistent with this interpretation, participants with non-zero minimization/denial scores reported significantly lower scores across all CTQ maltreatment subscales (Mann–Whitney tests: ps ≤ 0.013) (see Supplementary Table 2). Accordingly, as a robustness check against potential reporting bias, all subsequent analyses were repeated in the subsample with zero minimization/denial scores.

### Correlations

Table 1 presents the correlations among the main study variables (i.e., CTQ childhood maltreatment, RED food reward drive, and TFEQ disordered eating: Emotional eating, uncontrolled eating, and cognitive restraint).

The CTQ subscales were intercorrelated across a range from low to high, indicating that different forms of childhood maltreatment tended to co-occur. The largest associations were observed between emotional abuse and physical neglect and between physical abuse and physical neglect. Emotional abuse was also strongly related to physical abuse and emotional neglect.

CTQ scores showed small positive associations with RED food reward drive. Higher emotional abuse and emotional neglect were associated with higher food reward drive, with a smaller but significant association for physical abuse. Associations for sexual abuse and physical neglect were not significant.

In contrast to food reward drive, CTQ subscales were generally unrelated or negatively related to TEPS domain-general anticipatory pleasure. The clearest association was observed for emotional neglect, which was related to lower anticipatory pleasure. The remaining correlations between the CTQ and TEPS were close to zero. RED food reward drive and TEPS anticipatory pleasure showed a modest positive correlation.

RED food reward drive was strongly associated with uncontrolled eating and emotional eating but only weakly related to cognitive restraint. CTQ subscales showed small but consistent positive correlations with eating-related symptoms. Emotional abuse and emotional neglect were each associated with uncontrolled eating and emotional eating but not with cognitive restraint. Physical abuse showed small associations with both uncontrolled eating and emotional eating, with otherwise minimal relations. Physical neglect showed similar small associations with uncontrolled eating and emotional eating but was unrelated to cognitive restraint. Sexual abuse was not meaningfully related to any TFEQ subscale.

### Mediation models

Three path analysis models with a single mediator were tested using ordinary least squares regression (Table 3), one for each TFEQ subscale (uncontrolled eating, emotional eating, and cognitive restraint) as the outcome. In each model, the five CTQ subscales were entered simultaneously as predictors, with RED food reward drive as the mediator. First, the *a* paths were estimated by regressing RED food reward drive on the CTQ subscales. In this model, CTQ emotional abuse and emotional neglect subscales were positively associated with RED food reward drive, whereas the other CTQ subscales did not show clear unique associations with the RED when considered alongside the remaining subscales.

**Table 3:** Regression analysis: CTQ subscales, food reward drive (RED), and disordered eating symptoms (TFEQ) (*N* = 377).

Path	*β*	*B* (SE)	95% CI [L, U]	*p*-value	*R* ^2^
Path a: CTQ subscales → RED (mediator); all five predictors were entered simultaneously					
CTQ Emotional Abuse	0.170	0.356 (0.151)	[0.060, 0.652]	0.019	0.060
CTQ Physical Abuse	0.070	0.225 (0.213)	[−0.192, 0.642]	0.292	
CTQ Sexual Abuse	−0.039	−0.136 (0.186)	[−0.501, 0.229]	0.466	
CTQ Emotional Neglect	0.138	0.509 (0.219)	[0.080, 0.938]	0.021	
CTQ Physical Neglect	−0.107	−0.516 (0.325)	[−1.153, 0.121]	0.113	
Path b (RED) and direct effects (*c*′ paths): outcome = TFEQ Uncontrolled Eating (Y₁); all five CTQ subscales + RED were entered simultaneously					
RED Food Reward Drive	0.857	0.464 (0.014)	[0.437, 0.491]	<0.001	0.750
CTQ Emotional Abuse	−0.015	−0.017 (0.042)	[−0.099, 0.065]	0.683	
CTQ Physical Abuse	−0.024	−0.041 (0.060)	[−0.159, 0.077]	0.491	
CTQ Sexual Abuse	0.012	0.022 (0.052)	[−0.080, 0.124]	0.667	
CTQ Emotional Neglect	0.031	0.061 (0.062)	[−0.061, 0.183]	0.320	
CTQ Physical Neglect	0.071	0.186 (0.091)	[0.008, 0.364]	0.042	
Path b (RED) and direct effects (*c*′ paths): outcome = TFEQ Emotional Eating (Y₂); all five CTQ subscales + RED were entered simultaneously					
RED Food Reward Drive	0.592	0.156 (0.011)	[0.134, 0.178]	<0.001	0.379
CTQ Emotional Abuse	0.022	0.012 (0.033)	[−0.053, 0.077]	0.714	
CTQ Physical Abuse	−0.011	−0.010 (0.046)	[−0.100, 0.080]	0.833	
CTQ Sexual Abuse	0.009	0.008 (0.040)	[−0.070, 0.086]	0.843	
CTQ Emotional Neglect	0.047	0.045 (0.047)	[−0.047, 0.137]	0.339	
CTQ Physical Neglect	0.055	0.070 (0.070)	[−0.067, 0.207]	0.314	
Path b (RED) and direct effects (*c*′ paths): outcome = TFEQ Cognitive Restraint (Y₃); all five CTQ subscales + RED were entered simultaneously					
RED Food Reward Drive	0.217	0.104 (0.025)	[0.055, 0.153]	<0.001	0.051
CTQ Emotional Abuse	−0.041	−0.042 (0.073)	[−0.185, 0.101]	0.570	
CTQ Physical Abuse	−0.099	−0.152 (0.103)	[−0.354, 0.050]	0.140	
CTQ Sexual Abuse	0.026	0.043 (0.090)	[−0.133, 0.219]	0.636	
CTQ Emotional Neglect	−0.014	−0.026 (0.106)	[−0.234, 0.182]	0.811	
CTQ Physical Neglect	0.025	0.058 (0.157)	[−0.250, 0.366]	0.712	
Indirect effects (bootstrapped, 5,000 resamples)					
CTQ Emotional Abuse - TFEQ Uncontrolled Eating	0.165	–	[0.032, 0.317]	–	–
CTQ Physical Abuse - TFEQ Uncontrolled Eating	0.104	–	[−0.093, 0.320]	–	–
CTQ Sexual Abuse - TFEQ Uncontrolled Eating	−0.063	–	[−0.199, 0.075]	–	–
CTQ Emotional Neglect - TFEQ Uncontrolled Eating	0.236	–	[0.043, 0.428]	–	–
CTQ Physical Neglect - TFEQ Uncontrolled Eating	−0.239	–	[−0.562, 0.068]	–	–
CTQ Emotional Abuse - TFEQ Emotional Eating	0.056	–	[0.011, 0.106]	–	–
CTQ Physical Abuse - TFEQ Emotional Eating	0.035	–	[−0.030, 0.108]	–	–
CTQ Sexual Abuse - TFEQ Emotional Eating	−0.021	–	[−0.067, 0.026]	–	–
CTQ Emotional Neglect - TFEQ Emotional Eating	0.079	–	[0.010, 0.144]	–	–
CTQ Physical Neglect - TFEQ Emotional Eating	−0.081	–	[−0.189, 0.027]	–	–
CTQ Emotional Abuse - TFEQ Cognitive Restraint	0.037	–	[0.007, 0.079]	–	–
CTQ Physical Abuse - TFEQ Cognitive Restraint	0.023	–	[−0.021, 0.075]	–	–
CTQ Sexual Abuse - TFEQ Cognitive Restraint	−0.014	–	[−0.046, 0.018]	–	–
CTQ Emotional Neglect - TFEQ Cognitive Restraint	0.053	–	[0.007, 0.114]	–	–
CTQ Physical Neglect - TFEQ Cognitive Restraint	−0.054	–	[−0.138, 0.014]	–	–

Values are unstandardized (*β*) and standardized (β^*^) regression coefficients. SE, standard error. 95% CI = 95% confidence interval for the unstandardized coefficient. For indirect effects, the 95% CI is a percentile bootstrap CI (5,000 resamples); *p*-value and SE are not applicable. *R*^2^ is reported once per regression equation (a path equation: CTQ subscales → RED; *b*/*c′* path equation: CTQ subscales + RED → outcome). All predictors were entered simultaneously within each equation. CTQ, Childhood Trauma Questionnaire; RED, Reward-Based Eating Drive Scale; TFEQ, Three-Factor Eating Questionnaire.

Next, the *b* paths and direct effects (*c′* paths) were estimated in a single model by regressing each TFEQ subscale on the RED and CTQ subscales simultaneously. All variance inflation factors were low (VIFs = 1.06–2.06), indicating that multicollinearity was not a concern. In this model, the RED was a strong positive predictor of uncontrolled eating (*β* = 0.857) and emotional eating (*β* = 0.592) after controlling for all CTQ subscales (the *b* path) and a weaker but significant predictor of cognitive restraint (*β* = 0.217). The coefficients linking each CTQ subscale to each outcome in this model represent the direct effects (*c′* paths), that is, the association between each CTQ subscale and the outcome while holding the RED constant. With one exception (CTQ physical neglect showed a significant direct effect on uncontrolled eating), none of these coefficients was significant, suggesting that the association between CTQ and TFEQ subscales was largely accounted for by the indirect paths through the RED.

The total effects (*c* paths) of CTQ subscales on each TFEQ subscale were estimated in a separate model by regressing each outcome on CTQ subscales without including the RED. Indirect effects were computed for each CTQ subscale as the product of coefficients (*a* × *b*) and were evaluated using bootstrap confidence intervals (5,000 resamples).

Consistent with mediation, the indirect effects via the RED were statistically significant for CTQ emotional abuse and emotional neglect subscales across all three outcomes (uncontrolled eating, emotional eating, and cognitive restraint), as indicated by bootstrap 95% confidence intervals that excluded zero. In contrast, the indirect effects for CTQ physical abuse, sexual abuse, and physical neglect subscales were not statistically distinguishable from zero for any outcome.

With age, sex, SES, BMI, smoking status, current medication use, alcohol use, medical conditions, and mental health conditions included in the models, the indirect pathways via the RED remained significant for both CTQ emotional abuse and emotional neglect subscales across all three outcomes (see Supplementary Table 3).

*Sensitivity analysis in the subsample with CTQ minimization/denial scores of zero.* In participants with CTQ minimization/denial scores of zero (*n* = 276), higher emotional neglect remained significantly associated with higher RED scores, and higher RED scores were associated with higher scores across all three TFEQ subscales (see Supplementary Table 4). Only CTQ emotional neglect showed a significant indirect effect via the RED for uncontrolled eating, emotional eating, and cognitive restraint. The indirect effect for CTQ emotional abuse did not reach significance in this subsample, as its *a* path was no longer significant.

*Domain-general anticipatory pleasure as a mediator.* Among CTQ subscales, only emotional neglect was a significant predictor of domain-general anticipatory pleasure, with higher emotional neglect associated with lower anticipatory pleasure. TEPS Anticipatory Pleasure was a significant positive predictor of uncontrolled eating and emotional eating but not of cognitive restraint (see Supplementary Table 5). The indirect effects of CTQ emotional neglect via TEPS Anticipatory Pleasure were significant for uncontrolled eating and emotional eating but not for cognitive restraint. Indirect effects for the remaining CTQ subscales were not significant for any outcome.

## Discussion

The primary finding of this study is that food reward drive was supported as one of the mechanisms through which childhood emotional neglect predicts disordered eating symptoms. Specifically, higher levels of both childhood emotional neglect and emotional abuse were associated with higher food reward drive, and higher food reward drive was associated with increased disordered eating symptoms. This indirect effects were robust after controlling for sociodemographic characteristics (age, sex, and perceived socioeconomic status) and biomedical characteristics (e.g., BMI, smoking status, current medication use, alcohol use, medical conditions, and mental health conditions), as well as after controlling for the tendency to minimize childhood maltreatment experiences. The indirect effect of emotional abuse was also significant in the covariate-adjusted models but did not remain significant in the sensitivity analysis restricted to non-minimizers.

To the best of our knowledge, there is no prior evidence showing that higher food reward drive may explain the association between childhood emotional neglect and disordered eating. Rossi et al. (2025) examined food addiction as part of a pathway linking emotional maltreatment and dysregulated eating behavior. However, food addiction is a complex concept that captures both reward processes and disordered eating symptoms, and it is difficult to map onto current approaches to reward processes (National Institute of Mental Health, 2016; Carcone and Ruocco, 2017). The present results focus on specific processes contributing to food reward drive, such as heightened reactivity to palatable foods, persistent food-related thoughts, and reduced awareness of satiety cues, and show that these processes are enhanced in individuals with a history of childhood emotional neglect. Moreover, these increased levels may help explain higher levels of disordered eating. This has the advantage of identifying clear intervention targets that may help reduce disordered eating among individuals with a history of emotional neglect (Rebello and Greenway, 2016; Pasquale et al., 2024).

How might one explain the association between childhood emotional neglect and food reward drive? It is conceivable that, in the absence of adequate adult support for stress regulation, emotionally neglected children may resort to primary rewards such as food as a coping strategy. This hypothesis is supported by studies showing that childhood emotional neglect is associated with increased anxiety (Kennedy et al., 2007) and stress (Hund and Espelage, 2006) and that these negative emotional states may contribute to the pathway to disordered eating. We suggest that increased food reward drive is downstream of these negative emotions and reflects attempts to use food as a coping mechanism in the context of childhood emotional neglect (Rossi and Pesavento, 2025). This interpretation is consistent with evidence showing that emotion regulation is involved in the pathway between emotional neglect and disordered eating (Moulton et al., 2015). However, we argue that enhanced food reward drive represents a specific compensatory mechanism: it may serve coping functions while being specifically tied to food rather than other potential rewards. Food is typically readily available, whereas other rewards (e.g., social recognition) may require additional effort and may be particularly scarce in environments dominated by emotionally neglectful parents. Therefore, emotionally neglected children may specifically turn to food to cope with stress, and this pattern may stabilize over time, emerging in adulthood as increased food reward drive, as reflected in dispositional measures such as the RED.

A lingering question is why only emotional neglect—and not other forms of childhood adversity—showed a significant association with food reward drive. We propose that this connection reflects the chronic, pervasive nature of emotional neglect: unlike physical or sexual abuse, which are more episodic and can lead to coping responses such as avoidance or dissociation, emotional neglect persistently deprives children of social rewards, making food a particularly accessible and repeatedly used alternative that gradually consolidates into elevated food reward drive in adulthood. It is also worth noting that the majority of participants reported minimal exposure to physical abuse, sexual abuse, and physical neglect, leaving little variability in these scores and likely reducing statistical power. Consistent with our results, Vajda and Láng (2014) found that emotional abuse and neglect, but not physical or sexual abuse, differentiated eating disorder patients from healthy controls and were specifically linked to emotion regulation deficits.

Consistent with this specificity, the results of this study show that the associations between childhood emotional neglect and food reward drive and domain-general anticipatory pleasure are opposite in direction. Unlike the blunted reward processing typically observed following childhood maltreatment (Miu et al., 2017), maltreatment—particularly childhood emotional neglect— positively predicted food reward drive in this study. The moderate correlation between food reward drive and domain-general anticipatory pleasure (*r* = 0.27) underscores their distinctiveness as constructs. Furthermore, the indirect effect of childhood emotional neglect on disordered eating was larger and more consistent through food reward drive than through domain-general anticipatory pleasure. This domain-specific pattern may help explain the heterogeneity observed in meta-analyses of maltreatment and reward processing when food rewards are excluded (Oltean et al., 2023), and it suggests that future studies should systematically consider reward type (e.g., primary vs. secondary) (Delgado et al., 2006).

In the present study, food reward drive showed stronger positive associations with emotional eating and uncontrolled eating than with cognitive restraint, directly paralleling maltreatment’s pattern across these subscales. This symptom specificity reflects a shared hedonic component—excessive pleasure from food rewards—coupled with divergent regulatory styles: Disinhibition and loss of control in emotional and uncontrolled eating versus deliberate behavioral inhibition in cognitive restraint. These dynamics mirror the binge–restrictive spectrum of eating pathology, where binge-like behaviors reflect reward-driven loss of control and restrictive behaviors reflect active suppression (Wierenga et al., 2014; Pasquale et al., 2024). This distinction aligns with experimental evidence showing greater sensitivity to food cues among uncontrolled eaters, whereas restrained eaters show diminished responses (Hume et al., 2016; Coletta et al., 2009).

Several limitations should be considered when interpreting these findings. Although the sample was predominantly female (79.5%), sensitivity analyses showed that the main results were robust after controlling for sex, as well as a range of other sociodemographic and biomedical characteristics. The two-wave design limits the robustness of our conclusions regarding the direction of the observed associations, and future longitudinal studies should address this issue, particularly regarding the direction of the association between food reward drive and disordered eating symptoms. Another limitation is the exclusive reliance on self-report measures, which may have introduced potential method variance. Future studies should employ multimethod approaches, such as EEG/ERP measures and behavioral tasks assessing reward processes (Banica et al., 2022). Finally, the use of incentives for participation (course credit and prize draw entry) may have introduced motivational bias, which should be considered when generalizing these findings.

These findings have important clinical implications. Routinely assessing childhood emotional neglect in individuals who present with disordered eating may help identify those for whom food reward drive is a key underlying mechanism. In such cases, interventions directly targeting reward-driven eating, such as mindfulness-based approaches addressing food cue reactivity or cognitive–behavioral strategies aimed at reducing reliance on food as a coping mechanism for negative affect, may be particularly beneficial. Importantly, such interventions may need to extend beyond targeting eating behaviors to address disrupted emotion regulation processes that manifest as an elevated food reward drive, through which early emotional neglect exerts its lasting influence on disordered eating.

In summary, the present study establishes food reward drive as a key mechanism through which childhood emotional neglect increases vulnerability to disordered eating symptoms, particularly uncontrolled and emotional eating. By demonstrating the food-specific and domain-specific nature of this pathway, these findings advance our mechanistic understanding of childhood emotional neglect’s lasting effects on eating behavior.

## Data Availability

The original contributions presented in the study are included in the article/Supplementary material, further inquiries can be directed to the corresponding author.
