# Supplementary material for: Childhood adversity and disordered eating: the mediator role of food reward drive
Source: Front Psychol. 2026 Aug 3;17:1863415. doi: 10.3389/fpsyg.2026.1863415 (PMC13478131; doi:10.3389/fpsyg.2026.1863415)
Supplement: Supplementary file 1 [file Supplementary_File_1.docx]

**Supplementary Table 1**

Frequency of participants in childhood maltreatment categories

| Subscale | None/Minimal | Low to Moderate | Moderate to Severe | Severe to Extreme |
| --- | --- | --- | --- | --- |
| CTQ Emotional Abuse | 156 (41.4%) | 88 (23.3%) | 53 (14.1%) | 80 (21.2%) |
| CTQ Emotional Neglect | 207 (54.9%) | 166 (44.0%) | 4 (1.1%) | 0 (0.0%) |
| CTQ Physical Abuse | 286 (75.9%) | 34 (9.0%) | 30 (8.0%) | 27 (7.2%) |
| CTQ Physical Neglect | 290 (76.9%) | 45 (11.9%) | 30 (8.0%) | 12 (3.2%) |
| CTQ Sexual Abuse | 298 (79.0%) | 31 (8.2%) | 27 (7.2%) | 21 (5.6%) |

*Note:* Values are reported as frequency (%, calculated within subscale, may not sum to 100% due to rounding). Severity categories for each CTQ subscale were assigned using standard cutoffs (Bernstein, & Fink, 1998). Abbreviations: CTQ = Childhood Trauma Questionnaire.

**Supplementary Table 2**

Childhood maltreatment according to CTQ Minimization Denial scores

| CTQ subscale | CTQ Minimization Denial = 0 (n = 276) | CTQ Minimization Denial > 0 (n = 101) | Mann-Whitney *U* | p |
| --- | --- | --- | --- | --- |
| Emotional Abuse | 12.37 ± 0.32 | 7.28 ± 0.25 | 22299 | < .001 |
| Physical Abuse | 7.46 ± 0.23 | 5.68 ± 0.14 | 18188 | < .001 |
| Sexual Abuse | 6.3 ± 0.2 | 5.54 ± 0.24 | 15590 | 0.013 |
| Emotional Neglect | 10.01 ± 0.16 | 6.05 ± 0.17 | 24790 | < .001 |
| Physical Neglect | 7.07 ± 0.15 | 5.2 ± 0.06 | 22109 | < .001 |

*Note.* Values are reported as M ± SEM. Group differences were tested using two-tailed Mann–Whitney U tests due to positive skew in CTQ subscale distributions. Abbreviations: CTQ = Childhood Trauma Questionnaire.

**Supplementary Table 3**

Paths between childhood maltreatment, food reward drive and disordered eating symptoms, with covariates included in the model

| **CTQ subscale** | **Path a (CTQ → RED)** | | | | | **Path b (RED → outcome)** | | | | | | **Indirect effect** | |
| --- | --- | --- | --- | --- | --- | --- | --- | --- | --- | --- | --- | --- | --- |
|  | *B* | SE(*B*) | *β* | *p* | *R²* | *B* | SE(*B*) | *β* | *p* | *R²* |  | | |
| **Panel A. Uncontrolled Eating** | | | | | | | | | | | | | |
| Emotional Abuse | .290 | .153 | .091 | .013 | .166 | .456 | .015 | .843 | < .001 | .761 | .132* | | [.036, .232] |
| Physical Abuse | .258 | .207 | .074 | .132 |  | .456 | .015 | .843 | < .001 |  | .118 | | [-.033, .270] |
| Sexual Abuse | .003 | .180 | -.047 | .987 |  | .456 | .015 | .843 | < .001 |  | .001 | | [-.139, .138] |
| Emotional Neglect | .565 | .212 | .140 | .002 |  | .456 | .015 | .843 | < .001 |  | .256* | | [.099, .413] |
| Physical Neglect | .253 | .339 | -.074 | .364 |  | .456 | .015 | .843 | < .001 |  | .115 | | [-.121, .357] |
| **Panel B. Emotional Eating** | | | | | | | | | | | | | |
| Emotional Abuse | .290 | .153 | .091 | .013 | .166 | .148 | .012 | .551 | < .001 | .413 | .043* | | [.011, .077] |
| Physical Abuse | .258 | .207 | .074 | .132 |  | .148 | .012 | .551 | < .001 |  | .038 | | [-.012, .087] |
| Sexual Abuse | .003 | .180 | -.047 | .987 |  | .148 | .012 | .551 | < .001 |  | .000 | | [-.044, .049] |
| Emotional Neglect | .565 | .212 | .140 | .002 |  | .148 | .012 | .551 | < .001 |  | .083* | | [.031, .137] |
| Physical Neglect | .253 | .339 | -.074 | .364 |  | .148 | .012 | .551 | < .001 |  | .038 | | [-.042, .119] |
| **Panel C. Cognitive Restraint** | | | | | | | | | | | | | |
| Emotional Abuse | .290 | .153 | .091 | .013 | .166 | .085 | .026 | .170 | .001 | .144 | .025* | | [.005, .055] |
| Physical Abuse | .258 | .207 | .074 | .132 |  | .085 | .026 | .170 | .001 |  | .022 | | [-.005, .060] |
| Sexual Abuse | .003 | .180 | -.047 | .987 |  | .085 | .026 | .170 | .001 |  | .000 | | [-.024, .030] |
| Emotional Neglect | .565 | .212 | .140 | .002 |  | .085 | .026 | .170 | .001 |  | .048* | | [.012, .096] |
| Physical Neglect | .253 | .339 | -.074 | .364 |  | .085 | .026 | .170 | .001 |  | .021 | | [-.023, .076] |

*Note.* Covariates included age, sex, socioeconomic status at age 12 and current socioeconomic status, BMI, smoking, alcohol use, current medication, and current medical and mental health problems. Complete-case N used for this table was 376 (one case from the original N = 377 complete-case CTQ/REDT1/TFEQ dataset was excluded due to missingness on at least one covariate)*.* Values are unstandardized ordinary least squares regression coefficients. Standardized coefficients are reported as β. p values are two-tailed. R² is the model coefficient of determination (a-path model includes all five CTQ subscales and covariates; b-path model includes RED, all five CTQ subscales and covariates). Abbreviations: CTQ = Childhood Trauma Questionnaire; RED = Reward-based Eating Drive; TFEQ = Three-Factor Eating Questionnaire.

**Supplementary Table 4.**

Sensitivity Analysis: Paths between childhood maltreatment, food reward drive and disordered eating symptoms, in the subsample with zero scores on the CTQ Minimization/Denial (n = 276)

| **CTQ subscale** | **Path a (CTQ → RED)** | | | | | **Path b (RED → outcome)** | | | | | **Indirect effect** | |
| --- | --- | --- | --- | --- | --- | --- | --- | --- | --- | --- | --- | --- |
|  | *B* | SE(*B*) | *β* | *p* | *R²* | *B* | SE(*B*) | *β* | *p* | *R²* |  |  |
| **Panel A. Uncontrolled Eating** | | | | | | | | | | | | |
| Emotional Abuse | .253 | .161 | .123 | .117 | .044 | .461 | .018 | .851 | < .001 | .731 | .116 | [-.017, .268] |
| Physical Abuse | .255 | .221 | .089 | .251 |  | .461 | .018 | .851 | < .001 |  | .117 | [-.090, .328] |
| Sexual Abuse | -.073 | .202 | -.023 | .718 |  | .461 | .018 | .851 | < .001 |  | -.034 | [-.185, .128] |
| Emotional Neglect | .550 | .269 | .132 | .042 |  | .461 | .018 | .851 | < .001 |  | .253* | [.001, .478] |
| Physical Neglect | -.433 | .336 | -.097 | .199 |  | .461 | .018 | .851 | < .001 |  | -.199 | [-.510, .113] |
| **Panel B. Emotional Eating** | | | | | | | | | | | | |
| Emotional Abuse | .253 | .161 | .123 | .117 | .044 | .160 | .013 | .597 | < .001 | .372 | .041 | [-.008, .097] |
| Physical Abuse | .255 | .221 | .089 | .251 |  | .160 | .013 | .597 | < .001 |  | .041 | [-.032, .115] |
| Sexual Abuse | -.073 | .202 | -.023 | .718 |  | .160 | .013 | .597 | < .001 |  | -.012 | [-.065, .044] |
| Emotional Neglect | .550 | .269 | .132 | .042 |  | .160 | .013 | .597 | < .001 |  | .088* | [.001, .172] |
| Physical Neglect | -.433 | .336 | -.097 | .199 |  | .160 | .013 | .597 | < .001 |  | -.069 | [-.183, .042] |
| **Panel C. Cognitive Restraint** | | | | | | | | | | | | |
| Emotional Abuse | .253 | .161 | .123 | .117 | .044 | .124 | .029 | .257 | < .001 | .071 | .031 | [-.006, .082] |
| Physical Abuse | .255 | .221 | .089 | .251 |  | .124 | .029 | .257 | < .001 |  | .031 | [-.026, .094] |
| Sexual Abuse | -.073 | .202 | -.023 | .718 |  | .124 | .029 | .257 | < .001 |  | -.009 | [-.051, .035] |
| Emotional Neglect | .550 | .269 | .132 | .042 |  | .124 | .029 | .257 | < .001 |  | .068* | [.003, .150] |
| Physical Neglect | -.433 | .336 | -.097 | .199 |  | .124 | .029 | .257 | < .001 |  | -.053 | [-.152, .032] |

*Note.* Participants with CTQ Minimisation/Denial scores above zero (n = 101) were excluded, retaining only those who did not minimise or deny childhood maltreatment experiences. Values are unstandardized regression coefficients (B) with standard errors (SE(B)). Standardized coefficients are reported as β. p values are two-tailed. R² is the model coefficient of determination. Abbreviations: CTQ = Childhood Trauma Questionnaire; RED = Reward-based Eating Drive; TFEQ = Three-Factor Eating Questionnaire, UE = Uncontrolled Eating; EE = Emotional Eating; CR = Cognitive Restraint.

**Supplementary Table 5.**

Path Analysis with Domain-General Anticipatory Pleasure (TEPSap) as Mediator: CTQ Subscales Predicting Disordered Eating Symptoms (N = 377)

| **CTQ subscale** | **Path a (CTQ → TEPSap)** | | | | **Path b (TEPSap → outcome)** | | | | | | **Indirect effect (95% CI)** | | |
| --- | --- | --- | --- | --- | --- | --- | --- | --- | --- | --- | --- | --- | --- |
|  | *B* | SE(*B*) | *β* | *p* | → UE B | *p* | → EE B | *p* | → CR B | *p* | → UE | → EE | → CR |
| Emotional Abuse | .055 | .153 | .024 | .538 | .247 | < .001 | .099 | < .001 | .005 | .913 | .014 [-.040, .072] | .006 [-.016, .030] | .000 [-.008, .017] |
| Physical Abuse | .117 | .213 | .032 | .359 | .247 | < .001 | .099 | < .001 | .005 | .913 | .029 [-.036, .104] | .012 [-.014, .042] | .001 [-.020, .016] |
| Sexual Abuse | -.039 | .186 | -.010 | .729 | .247 | < .001 | .099 | < .001 | .005 | .913 | -.010 [-.065, .051] | -.004 [-.026, .022] | .000 [-.012, .012] |
| Emotional Neglect | -.422 | .219 | -.101 | .001 | .247 | < .001 | .099 | < .001 | .005 | .913 | -.104* [-.203, -.031] | -.042* [-.083, -.011] | -.002 [-.050, .035] |
| Physical Neglect | -.053 | .325 | -.010 | .786 | .247 | < .001 | .099 | < .001 | .005 | .913 | -.013 [-.116, .078] | -.005 [-.051, .031] | .000 [-.023, .019] |

*Note.* The b path for TEPSap → Cognitive Restraint was not significant (B = .005, p = .913), explaining the absence of significant indirect effects for Cognitive Restraint in all rows. Values are unstandardized regression coefficients (B) with standard errors (SE(B)). Standardized coefficients are reported as β. p values are two-tailed. The b path B values are shown for each outcome separately (same for all predictor rows within each outcome column). Abbreviations: CTQ = Childhood Trauma Questionnaire; TEPSap = Temporal Experience of Pleasure Scale – anticipatory pleasure subscale; TFEQ = Three-Factor Eating Questionnaire, UE = Uncontrolled Eating; EE = Emotional Eating; CR = Cognitive Restraint.

**Supplementary Table 6**

Comparison of T3 Completers and Non-Completers on Key T2 Variables

| **Variable** | **T3 Completers M (SD)** | **T3 Non-completers M (SD)** | **t** | **df** | **p** | **d** |
| --- | --- | --- | --- | --- | --- | --- |
| CTQ Emotional Abuse | 11.21 (5.10) | 10.29 (5.50) | 1.44 | 375 | .152 | 0.17 |
| CTQ Physical Abuse | 6.95 (3.34) | 7.11 (3.65) | -0.38 | 375 | .708 | -0.05 |
| CTQ Sexual Abuse | 6.13 (3.26) | 5.98 (2.73) | 0.40 | 375 | .688 | 0.05 |
| CTQ Emotional Neglect | 9.15 (2.90) | 8.24 (3.05) | 2.52 | 375 | .012 | 0.31 |
| CTQ Physical Neglect | 6.62 (2.29) | 6.39 (2.09) | 0.81 | 375 | .420 | 0.10 |
| RED | 28.73 (10.91) | 28.60 (10.89) | 0.10 | 375 | .920 | 0.01 |
| TFEQ Uncontrolled Eating | 17.69 (5.74) | 17.89 (6.41) | -0.27 | 375 | .784 | -0.03 |
| TFEQ Emotional Eating | 6.10 (2.83) | 6.45 (3.00) | -0.99 | 375 | .325 | -0.12 |
| TFEQ Cognitive Restraint | 15.27 (5.16) | 16.94 (5.24) | -2.60 | 375 | .010 | -0.32 |
| TEPS Anticipatory Pleasure | 31.65 (6.10) | 32.50 (7.41) | -1.08 | 375 | .282 | -0.13 |
| BMI | 23.28 (4.98) | 22.77 (3.83) | 0.87 | 375 | .385 | 0.12 |
| Age | 27.58 (11.26) | 25.83 (10.64) | 1.26 | 374 | .207 | 0.16 |
| Sex (% female) | 87.4% | 82.1% | — | — | .137 | — |

*Note.* Values are reported as M (SD) for continuous variables. Sex is reported as percentage female; group differences for sex were assessed using a chi-square test. d = Cohen's d. CTQ = Childhood Trauma Questionnaire; RED = Reward-based Eating Drive; TFEQ = Three-Factor Eating Questionnaire; TEPS = Temporal Experience of Pleasure Scale; BMI = body mass index.
